# The Receptor-Bound Guanylyl Cyclase DAF-11 Is the Mediator of Hydrogen Peroxide-Induced Cgmp Increase in *Caenorhabditis elegans*

**DOI:** 10.1371/journal.pone.0072569

**Published:** 2013-08-27

**Authors:** Ulrike Beckert, Wen Yih Aw, Heike Burhenne, Lisa Försterling, Volkhard Kaever, Lisa Timmons, Roland Seifert

**Affiliations:** 1 Institute of Pharmacology, Hannover Medical School, Hannover, Germany; 2 Department of Molecular Biosciences, University of Kansas, Lawrence, Kansas, United States of America; 3 Research Core Unit Mass Spectrometry-Metabolomics, Hannover Medical School, Hannover, Germany; University of Bonn, Germany

## Abstract

Adenosine 3′, 5′-cyclic monophosphate (cAMP) and guanosine 3′, 5′-cyclic monophosphate (cGMP) are well-studied second messengers that transmit extracellular signals into mammalian cells, with conserved functions in various other species such as *Caenorhabditis elegans* (*C. elegans*). cAMP is generated by adenylyl cyclases, and cGMP is generated by guanylyl cyclases, respectively. Studies using *C. elegans* have revealed additional roles for cGMP signaling in lifespan extension. For example, mutants lacking the function of a specific receptor-bound guanylyl cyclase, DAF-11, have an increased life expectancy. While the *daf-11* phenotype has been attributed to reductions in intracellular cGMP concentrations, the actual content of cyclic nucleotides has not been biochemically determined in this system. Similar assumptions were made in studies using phosphodiesterase loss-of-function mutants or using adenylyl cyclase overexpressing mutants. In the present study, cyclic nucleotide regulation in *C. elegans* was studied by establishing a special nematode protocol for the simultaneous detection and quantitation of cyclic nucleotides. We also examined the influence of reactive oxygen species (ROS) on cyclic nucleotide metabolism and lifespan in *C. elegans* using highly specific HPLC-coupled tandem mass-spectrometry and behavioral assays. Here, we show that the relation between cGMP and survival is more complex than previously appreciated.

## Introduction

The first description of guanosine 3′, 5′-cyclic monophosphate (cGMP) as a biological substance can be dated back to 1963 [1]. cGMP is now a well-established second messenger, like the earlier identified adenosine 3′, 5′-cyclic monophosphate (cAMP) [2], [3]. These cyclic nucleotides (cNMPs) are generated by the ATP- and GTP-converting adenylyl- and guanylyl cyclase, respectively [1], [2], [3], [4], [5]. cAMP and cGMP transmit extracellular signals into mammalian cells, with conserved functions in various other species such as *Caenorhabditis elegans.* In mammals, two classes of cGMP-forming enzymes have been identified: the soluble, nitric oxide (NO)-dependent guanylyl cyclase (sGC) and the particulate (membrane-bound) guanylyl cyclases (pGC) that are activated by natriuretic peptides [4], [5], [6], [7], [8] (reviewed in [8]). cGMP has an impact on various physiological processes such as smooth muscle relaxation, platelet aggregation and phototransduction. Not surprisingly, the cGMP signaling cascade has become an important pharmacological target, with successful regimens developed for the therapy of heart failure, (pulmonary) arterial hypertension and erectile dysfunction [9], [10].

*C. elegans* was first established as laboratory model organism by Sydney Brenner in 1974 [11]. It has become a favored model organism in genetic studies due, in part, to the completion of its genome sequence in 1998. Forty-two percent of the approximately 20,000 predicted *C. elegans* genes have homology to human genes [12], including those encoding guanylyl cyclases. The genome of *C. elegans* harbors 32 genes with similarity to guanylyl cyclases. These include 25 membrane-bound, receptor-like guanylyl cyclases and 7 cytosolic guanylyl cyclases (gcy-31–gcy-37) [13]. Two of the receptor-bound guanylyl cyclase genes, *daf-11* and *odr-1,* are expressed in olfactory and pheromone-sensing neurons and act downstream of G-protein-coupled receptors. *daf-11* is expressed in the ciliated ASI, ASJ, AWC, AWB, ASK neurons where it is involved in olfactory and pheromone sensing and behavior [14], [15]. *daf-11* plays a non-autonomous role in chemotaxis in ciliated ASE neurons [14], and *daf-11* regulates *dauer*-formation and recovery.

Recent studies have revealed roles for *daf-11* in *C. elegans* aging and oxidative stress response pathways, leading to the conclusion that cGMP signaling in *C. elegans* is interconnected with the insulin/IGF-1/DAF-16 signaling pathway implicated in longevity and stress resistance [16], [17]. *daf-11* mutants display enhanced longevity that is dependent upon DAF-16/FOXO, a phenotype that is similar to that observed in *gpa-3* mutants with defects in G-protein signaling in sensory neurons [16]. Several lines of evidence implicate altered cGMP pools in these GPA-3-related biological functions: *a*) Constitutively activated *gpa-3* mutants display an increased tolerance to oxidants and an increased lifespan. *b*) *daf-11* mutants defective in guanylyl cyclase display similar phenotypes [16]. *c*) activation of the G-protein GPA-3 leads to an increase in *pde-1* and *pde-5* mRNA levels [16]. As these genes encode phosphodiesterase enzymes that degrade cNMPs, the cGMP pool can be predicted to decrease as a result [16]. *d*) *pde-1* and *pde-5* mRNA levels also increase when animals are exposed to cGMP; similarly, transcriptional upregulation for these *pde* genes is also observed when *daf-11* mutants are exposed to cGMP [16]. *e*) *daf-11* mutants exposed to *dauer*-inducing environmental conditions display suppressed DAF-28/insulin levels, a finding which provides an interesting link between cGMP production and the insulin-related longevity and stress-response pathway [17].

Until now, it has not been shown biochemically that the *daf-11* loss of function mutant indeed contains less cGMP than wild-type animals. Moreover, *C. elegans* studies using phosphodiesterase loss-of-function mutants in phototransduction experiments [18] or adenylyl cyclase-overexpressing mutants in axon regeneration experiments [19] also assumed higher levels of cNMPs. While it may seem logical to assume that removal of one of the thirty-two guanylyl cyclase enzymes from *C. elegans* might result in lower intracellular levels of cGMP, studies from mammalian cardiac tissue highlight the complexity of cNMP metabolism. The intracellular concentrations of a given cNMP is a reflection of the balance between its synthesis its degradation, coordinated cross-talk between the cAMP and cGMP metabolic pathways [20], and of allosteric regulation of enzyme function [21]. From the studies in mammalian cells, we can anticipate that the six different cAMP and cGMP phosphodiesterases in *C. elegans* are likely to be allosterically regulated by cGMP and cAMP, respectively, and subject to competitive inhibition as well. Thus, removal of the catalytic enzyme may produce unanticipated outcomes in cNMP concentrations. For example, it is feasible that a reduced level of cGMP synthesis might lead to an increase in intracellular cAMP, due to lack of allosteric inhibition by the corresponding phosphodiesterase. In such a situation, cAMP might unexpectedly be the effector molecule mediating biological functions. The relatively simpler *C. elegans* genetics system affords particular advantages for the analysis of such a complicated array of regulatory connections. However, even though *C. elegans* has fewer tissues and fewer gene splice variants than mammalian systems, there are inherent complexities in an organism with thirty-two guanylyl cyclases, four adenylyl cyclases, and six phosphodiesterase genes that are incompletely characterized biochemically. In the present study, we established a highly specific HPLC-coupled tandem mass-spectrometry method for the simultaneous detection and quantitation of cAMP and cGMP in *C. elegans*. We used the nematode as a model organism to examine the influence of reactive oxygen species (ROS) on cNMP metabolism and lifespan. The assays were directed towards a better understanding of the roles of the guanylyl cyclase *daf-11,* cGMP phosphodiesterase, and cGMP-dependent protein kinase within the oxidative stress response of the nematode.

## Materials and Methods

### Materials

cGMP and cAMP were supplied by Biolog (Bremen, Germany) in a purity of >99%. 2′-Deoxy-5-fluorouridine (FUDR), forskolin (FSK) and peptone were purchased from Sigma-Aldrich (Seelze, Germany). Ammonium acetate was purchased from Fluka (Buchs, Switzerland). Magnesium sulfate, di-potassium hydrogen phosphate, potassium di-hydrogen phosphate, di-sodium hydrogen phosphate, sodium di-hydrogen phosphate, calcium chloride, sodium chloride, sodium ammonium mono hydrogen phosphate, H_2_O_2_ 30% (v/v) and cholesterol were obtained from Merck (Darmstadt, Germany). Sodium hypochlorite (NaOCl) 12% (v/v) was purchased from Hedinger (Stuttgart, Germany). HPLC grade acetonitrile, methanol and water were supplied by Baker (Deventer, The Netherlands) and acetic acid was purchased from Riedel de Häen (Seelze, Germany). Roti® Quant Bradford protein quantitation reagent, ethanol and sodium hydroxide were supplied by Roth (Karlsruhe, Germany). Taq Man probes and Taq Man Gene Expression Mastermix were purchased from Applied Biosystems (Carlsbad, CA, USA). 5x Real Time buffer was purchased from Fermentas (St. Leon-Rot, Germany). Bactoagar and LB Medium Difco™ Luria Bertani Broth Miller were supplied by Beckton Dickinson (Franklin Lakes, NJ, USA). Tenofovir was obtained from National Institute of Health AIDS Research and Reference Program, Division AIDS (Bethesda, MD, USA).

### Strains

The C. elegans strains used in this study were N2 Bristol (wild-type), KG522 (acy-1 (md1756)III), KG744 (pde-4(ce268)II), DR47 (daf-11(m47)V), MT1074(egl-4/pkg-1(n479)IV) and TQ1828 (pde-1(nj57), pde-5(nj49) I, pde-3(nj59) II, pde-2(tm3098)III) (Table S1) and were, as well as the food strain E. coli OP50, provided by the Caenorhabditis elegans Genetics Center which is funded by NIH Office of Research Infrastructure Programs (P40 OD010440) (University of Minnesota, MN, USA www.cbs.umn.edu). For Table S1 we provide a supplementary reference list for characterization of C. elegans strains. Maintenance of C. elegans was performed as described [22].

### Lifespan Analysis

Lifespan assays were performed at 22°C on normal growth medium plates supplemented with 0.05 mg/ml FUDR (5- fluorodeoxyuracil). FUDR prevented progeny overgrowth and thus mixed generations. Synchronized populations were obtained by picking L1 larvae and transferred to the FUDR plates on their first day of adulthood (30–40 per plate, 3 plates each assay). The number of worms was counted every 1–2 days until death. Worms were counted dead when they did not respond to gentle prodding at head and tail and no pharyngeal pumping could be observed. Animals that crawled off the plate were excluded at the time of the event, which allowed those worms to be incorporated in the data set until the censor date and no loss of data occurred. Lifespan counting was performed with a Nikon SMZ745T binocular microscope (Nikon Europe, Düsseldorf, Germany).

### Stress Response Assay in the Presence of 10 mM H_2_O_2_

Stress resistance assays were performed at 22°C in a 96-well plate using each time freshly prepared 10 mM H_2_O_2_ as oxidative agent, as established by Wang et al. 2006 [23]. Additional dose-finding assays with *C. elegans* wild-type were performed (Figure S5) in order to identify the suitable concentration for a time-dependent stress assay setting. Synchronized populations were obtained by picking L1 larvae and transfer to a 1.5 ml Eppendorf tube containing 300 µl of M9 buffer. For one assay, about 30–40 worms were carefully distributed into the wells of the 96-well plate (6–10 worms/well) containing 50 µl of 10 mM H_2_O_2_. Animals were tapped every 15–30 min and scored as dead when they did not respond to the prodding with a platinum pick. Animals whose cuticle bursted directly after placing them in 10 mM H_2_0_2_ due to physical stress of moving them, were excluded from the analysis by the Kaplan-Meier-methodology. There was no evidence for strain-specific cuticle disruptions induced by H_2_O_2_. Lifespan counting was performed under a Nikon SMZ745T binocular microscope (Nikon Europe, Düsseldorf, Germany).

### Forskolin Incubation

Five hundred to 1000 synchronized adult (72 h old) wild-type hermaphrodites were washed with 1 ml M9 buffer into a 1.5 ml Eppendorf tube. When the animals were settled down in the tube, the buffer was aspirated and a mixture of *E. coli OP50* in M9 buffer and forskolin, a direct activator of adenlylyl cyclases [24], with a final concentration of 50 µM was added [19]. Higher concentrations of forskolin had no effect on cNMP concentrations of *C. elegans*. The cause may have been the precipitation of forskolin in higer concentrations due to the poor solubility in water/DMSO (solubility of forskolin 5 mg/ml 100% DMSO). The bacteria were needed for a better ingestion of forskolin. After 1 h, 2 h and 4 h the animals were processed for cNMP extraction and analysis.

### Extraction of cNMPs in *C. elegans*

Per sample 500–1000 synchronized 72 h old adult hermaphrodites were washed with 1 ml M9 buffer into a 1.5 ml safe-lock Eppendorf tube. The animals were washed six times with 1 ml M9 buffer on ice in order to remove residual *E. coli OP50*. After the last washing step the worms were incubated in M9 buffer for 20 min to allow for digestion of the remaining bacteria in their gut followed by a last washing step with 1 ml M9 buffer. The supernatant was carefully removed and 300 µl of ice-cold extraction medium (acetonitrile/methanol/water, 2/2/1 v/v/v) with 25 ng/ml tenofovir (TNF) as internal standard was added. Immediately, the sample was frozen in liquid nitrogen for 30 s in order to stop cNMP metabolism in the animals, followed by 60 s of incubation in a 37°C water bath. This freeze-thaw cycle was repeated 6 times. Subsequently, the sample was heated to 98°C for 20 min, cooled down on ice and centrifuged at 20,000×g, and 4°C for 10 min. The supernatant fluid was transferred into a new 2 ml Eppendorf tube. The pellet of dead animals was extracted two more times with 400 µl extraction medium for 15 min on ice and the supernatants were combined. The fluid was evaporated under a gentle nitrogen stream at 40°C. The residue was dissolved in water and analyzed. For determination of the protein content, the cell pellet was completely dried and resolved in 0.1 M NaOH at 95°C for 10 min. One hundred µl of the solution was used in a Bradford protein assay. For cNMP assessments of animals exposed to H_2_O_2_, per sample 500–1000 synchronized 72 h old adult hermaphrodites were washed with 1 ml M9 buffer into a 1.5 ml safe-lock Eppendorf tube. When the animals were settled, the M9 buffer was carefully removed and 200 µl of 10 mM H_2_O_2_ solution added. The worms were incubated at 22°C for indicated time points: 15 min, 30 min, 60 min, 90 min and 120 min. After the incubation time the H_2_O_2_ solution was removed and the animals washed with M9 buffer. Subsequently, the samples were treated as above.

### Quantitation of cNMPs by HPLC-MS/MS

The analysis of cNMP concentrations was performed *via* HPLC-MS/MS. HPLC separation was performed on an Agilent 1100 series (Waldbronn, Germany) equipped with a binary pump system. A Zorbax Eclipse XDB-C18 column (50×4.6 mm, 1.8 µm particle size, Agilent Technologies, CA, USA) was used as stationary phase for analyte separation. The binary pump system supplied two eluents for chromatographic analysis, eluent A (3/97 (v/v) methanol/H_2_O +50 mM NH_4_Oac +0.1% (v/v) acetic acid) and eluent B (97/3 (v/v) methanol/H_2_O +50 mM NH_4_Oac +0.1% (v/v) acetic acid). Parameters of the flow rates are documented in Table 1. Analyte detection was conducted on the sensitive Qtrap 5500 triple quadrupole mass spectrometer (ABSCIEX, Foster City, CA, USA) using selected reaction monitoring (SRM) analysis in positive ionization mode. Nitrogen was used as collision gas for this purpose. The parameters of the HPLC-MS/MS fragmentation and retention times are specified in Table 2. The mass spectrometer parameters were as follows: ion source voltage of 5,500 V; ion source temperature of 600°C; curtain gas of 30 psi and collision gas of 9 psi. cNMP concentration in samples was quantified using the calibration curve obtained by analysis of known amounts of pure cNMPs. Chromatographic data was collected and analyzed using Analyst 1.5.1. TF software.

**Table 1:** Parameters of flow rates and gradients.

Total time	Flow rate	A [%]	B [%]
0 min	0.4 µl/min	100	0
5 min	0.4 µl/min	50	50
5.1 min	0.4 µl/min	100	0
8 min	0.4 µl/min	100	0

**Table 2:** Detection and quantitation parameters of the quantification of cNMPs and IS TNF by HPLC-MS/MS.

	*cAMP*	*cCMP*	*cGMP*	*cUMP*	*tenofovir*
[M+H]^+^ [m/z]	330.0	306.0	346.0	307.0	288.0
Quantifier [m/z]	135.9	112.0	151.9	96.9	176.0
Qualifier [m/z]	312.0	95.1	135.0	112.9	270.0
Ratio quantifier/qualifier	1∶5.2	1∶4.0	1∶2.5	1∶1.9	1∶2.2
Retention time	6.0	4.0	5.5	5.0	5.8

[M+H]^+^: protonated molecule mass, [m/z]: mass per charge, IS: internal standard, TNF: tenofovir, HPLC-MS/MS: high performance liquid chromatography coupled tandem mass spectrometry.

### Validation of cNMP Quantitation by HPLC-MS/MS

A control set of cNMP samples (cAMP, cCMP, cGMP and cUMP) was prepared in order to validate the cNMP quantitation. One control set was prepared on five different days, of which five technical replicates were measured. The mean of the daily concentrations was taken resulting in the calculation of the mean of five days which is presented in Table 3 (interday precision). The intraday precision was calculated by measuring one set of controls per day with five technical replicates. The mean is depicted in Table 3. Additionally, the operator precision was determined. For this purpose, on one day, five control sets were prepared and measured one time. The mean is specified in Table 3. This validation resulted in a linear range of 0.04–160 pmol/sample for cAMP and 0.07–250 pmol/sample cCMP, cGMP and cUMP (table 4). The lower limits of quantitation (LLOQ, accuracy<20%) and the limits of detection (LOD, signal-to-noise>5) are depicted in Table 4 for each cyclic nucleotide.

**Table 3:** Validation of cNMP HPLC-MS/MS methodology.

	cAMP			cCMP			cGMP			cUMP		
	[pmol/sample]		CV [%]	[pmol/sample]		CV [%]	[pmol/sample]		CV [%]	[pmol/sample]		CV [%]
***Interday precision***												
***QC low***	0.105±0.004		4.1	0.154±0.01		8.4	0.178±0.01		6.3	1.59±0.05		3.1
***QC medium***	3.13±0.14		4.5	5.17±0.16		3.2	4.98±0.18		3.6	19.16±0.84		4.4
***QC high***	98.7±1.87		1.9	199.8±6.1		3.1	204±6.1		3.0	216.1±2.17		1.0
***Intraday precision***												
***QC low***	0.104±0.01		8.6	0.153±0.01		4.1	0.167±0.01		6.9	1.55±0.051		3.3
***QC medium***	2.95±0.06		1.9	4.92±0.07		1.5	4.78±0.13		2.8	18.08±2.58		2.6
***QC high***	97.4±2.35		2.41	202.4±9.23		4.6	206.4±16.5		8.0	215±7.6		3.5
***Operator precision***												
***QC low***	0.094±0.007	7.8		0.153±0.01	5.0		0.175±0.01	1.8		1.28±0.041	3.3	
***QC medium***	3.12±0.18	5.8		5.1±0.39	7.6		4.86±0.26	6.5		18.2±1.3	7.4	
***QC high***	98.7±3.7	3.8		191.2±5.5	2.9		206.6±7.9	4.6		177.2±8.2	4.7	

CV: coefficient of variation, HPLC-MS/MS: high performance liquid chromatography coupled tandem mass spectrometry, QC: quality control.

**Table 4:** Linear range, LLOQ and LOD of cNMP methodology.

	cAMP [pmol/sample]	cCMP [pmol/sample]	cGMP [pmol/sample]	cUMP [pmol/sample]
**Linear Range**	0.04–160	0.07–250	0.07–250	0.4–250
**LLOQ**	0.04	0.07	0.07	0.4
**LOD**	0.02	0.013	0.03	0.16

LLOQ: lower limit of quantitation, accuracy<20%, LOD: limit of detection, signal-to-noise>5.

### RNA Isolation

For RNA isolation of *C. elegans*, 500–1000 animals were pelleted in a 1.5 ml safe-seal Eppendorf tube and washed 6 times with M9 buffer. The buffer was completely removed and the animals were frozen immediately in liquid nitrogen. Subsequently, room-temperature RA1/β-mercaptoethanol solution (350 µl/3.5 µl) of the Nucleo Spin II-RNA Kit (Macherey & Nagel, Düren, Germany) was added to the pellet and frozen down at −80°C until use. The RNA extraction was performed with the Nucleo Spin II-RNA Kit (Macherey & Nagel, Düren, Germany), following the manufacturer’s instructions. The RNA concentrations were determined by NanoDrop (Thermo Scientific, Wilmington, DE, USA). Total RNA (1 µg per sample) was taken for first strand cDNA synthesis with MLVRT (Life Technologies, Darmstadt, Germany). cDNA content was assessed by NanoDrop (Thermo Scientific, Wilmington, DE, USA).

### Quantitative RT-PCR

A quantitative RT-PCR using Taqman probes was performed in order to quantify the expression of distinct genes. First, RNA isolation was performed followed by a reverse transcriptase reaction. In order to conduct the PCR 2 µl of cDNA were pipetted into the well of a 96-well plate and the following Taqman probes were added: Ce02425242_m1 (pde-1), Ce02450556_m1 (pde-2), Ce02421830_m1 (pde-3), Ce02421841_m1 (pde-4), Ce2417438_g1 (pde-5), Ce02413958_m1 (pde-6), Ce2481960_m1 (daf-11), Ce02407231_m1 (pkg-1). RT-PCR reactions were heat-started with 10 min at 95°C followed by 40 amplification cycles of 15 s at 95°C and 60 s at 60°C using TaqMan Universal Master Mix (Life Technologies) and a Step One Plus thermocycler (Applied Biosystems, Darmstadt, Germany). The data were quantified using the comparative Ct (ΔΔCt) method, taken into account the different amplification efficiencies for the different genes in the formula of ΔCt [25]. Data were normalized to the geometric mean of two housekeeping genes. As reference genes pmp-3 (putative ABC transporter) and Y45F10D.4 (putative iron-sulfur cluster enzyme) were used according to [26], [27], whose expression levels were stable during the assays.

### Statistics

Data are presented as means ± SEM or SD and are based on 3–12 independent experiments. GraphPad Prism software version 5.01 (San Diego, CA, USA) was used for calculation of means, SEM and SD. Lifespan analysis was analyzed *via* the Meier-Kaplan survival estimator and survival distributions were compared by Log-rank test (Mantel-Cox test). P-values were calculated by means of ANOVA Bonferroni’s multiple comparison test with ***: p-value≤0.001, **: p-value≤0.01, and *: p-value≤0.05. P-values≤0.05 were considered significant.

## Results

### An HPLC-MS/MS Assay for cAMP and cGMP in *C. elegans*

Wild-type animals were synchronized by bleaching and 72 h old adult hermaphrodites were freeze-cracked to break their cuticle in order to prepare the extracts. As proof-of-principle for these applications *in C. elegans*, we first quantified cAMP and cGMP levels in wild-type and mutant *C. elegans* strains. We compared the cNMP levels in wild-type adults (N2), *pde-4(ce268) II*, a semi-dominant, loss of function mutant, and a *pde-1(nj57) pde-5(nj49) I; pde-3(nj59) II; pde-2(tm3098) III* quadruple loss of function mutant (*pde-1,2,3,5*). PDE-4 is suggested to be a cAMP-degrading phosphodiesterase in *C. elegans* and PDE-1 and PDE-5 are postulated to degrade cNMPs, with a preference for cGMP [28].

**Figure 1:**
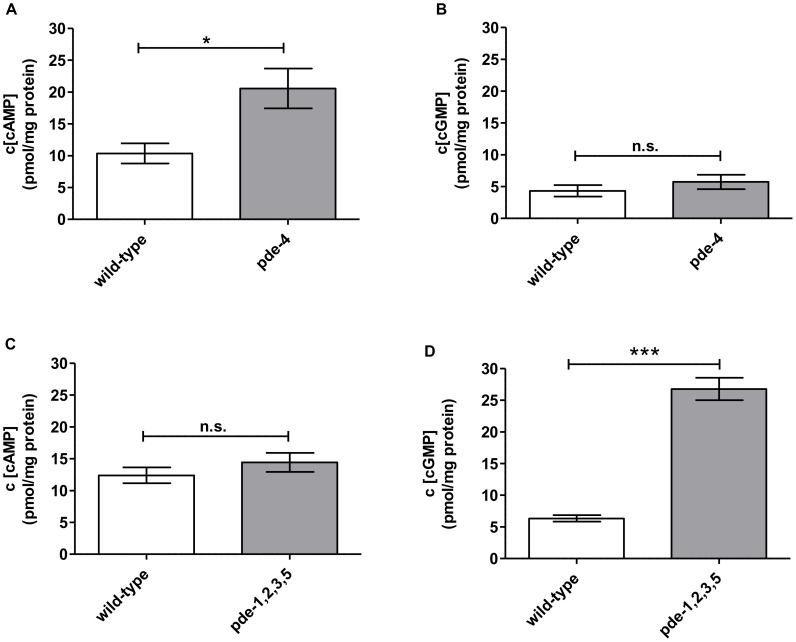
cAMP and cGMP concentrations in wild-type vs. *pde-4* (A, B) and wild-type vs. *pde-1,2,3,5* (C, D). Animals were grown on NGM plates at 22°C and harvested after they reached adulthood. N2: wild-type; *pde-4* mutant: cAMP degrading PDE; *pde-1,2,3,5* mutant: mainly cGMP degrading PDEs. **A, C** display basal cAMP and **B,D** basal cGMP concentrations. Values represent means ± SEM of at least three independent experiments. P-values were calculated by means of one-way ANOVA with Bonferroni’s multiple comparison test with *: p-value≤0.05, ***: p-value≤0.001, n.s.: not significant.

**Figure 2:**
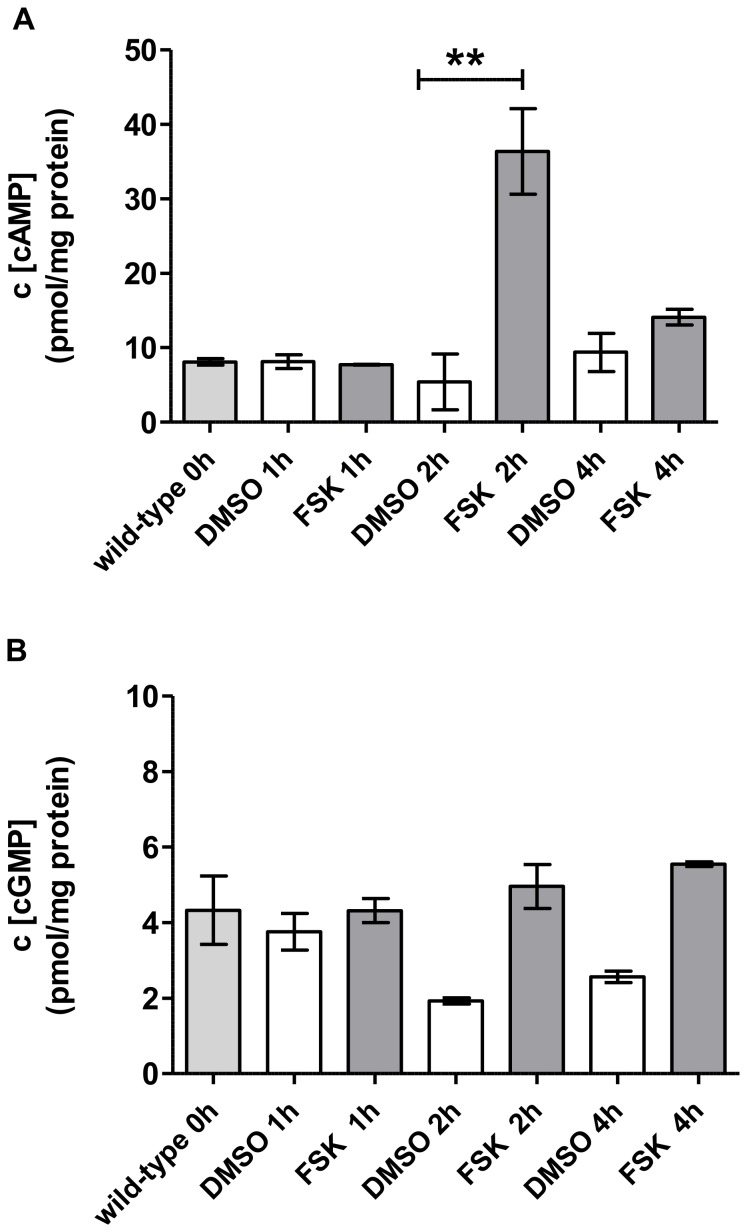
Effect of forskolin (FSK) on cAMP (A) and cGMP (B) concentrations in *C. elegans*. 3-day-old, adult animals were incubated with 50 µM FSK for the indicated times. After 1 h, 2 h or 4 h, the animals were washed with M9 buffer and processed for HPLC-MS/MS quantitation. Wild-type 0 h represents the untreated control. Values represent means ± SEM of three independent experiments in pmol/mg protein. Please note the different y-axes of panels A and B. P-values were calculated by means of one-way ANOVA with Bonferroni’s multiple comparison test with **: p-value≤0.005. The cGMP values are not significant according to the analysis by one-way ANOVA with Bonferroni’s multiple comparison test, the DMSO values decreased, but the cGMP values almost remained on untreated wild-type level (B).

Compared to wild-type animals, the *pde-4* mutants showed significantly higher concentrations of cAMP, a 2-fold increase, but the cGMP level remained similar to wild-type levels (Figure 1 A, B). In contrast, no change in cAMP levels were observed in the *pde-1,2,3,5* quadruple mutant compared to wild-type, while the animals displayed a strong increase in cGMP concentration (Figure 1 C, D). From these results we can infer that these two mutants indeed are defective in degradation of cAMP or cGMP, respectively, and that the differences in overall cNMP concentrations in whole animals can be measured. The cAMP concentrations of an adenylyl cyclase gain-of-function mutant (*acy-1-gf*) were not significantly changed in comparison to wild-type animals (Figure S1). We further tested our ability to quantify cAMP and cGMP in the nematode by analyzing forskolin-treated animals. Wild-type animals were incubated in a mixture of *E. coli OP50* food and forskolin, a direct activator of adenylyl cyclases [19], with a final concentration of 50 µM for 1 h, 2 h or 4 h. Only after 2 h, a 3.5-fold increase of cAMP was detected whereas the cGMP concentration was not affected by the addition of forskolin (Figure 2). Forskolin needed to be ingested by the animals which could explain the increase of cAMP not until 2 h. Rapid metabolic inactivation of forskolin by the animals may explain the only transient cAMP increase, i.e. after 4 h, the forskolin effect was only minimal. The forskolin results support the findings in Figure 1 and help to validate our methodology to detect and measure cAMP and cGMP in *C. elegans* in addition to the validation methodology described in Materials in Methods (Table 3). We could define a linear range of 0.04–160 pmol/sample for cAMP and 0.07–250 pmol/sample for cCMP, cGMP and cUMP (Table 4). The lower limit of detection was found to be 0.02 pmol/sample for cAMP, 0.013 pmol/sample for cCMP, 0.03 pmol/sample for cGMP and 0.16 pmol/sample for cUMP (Table 4). Cyclic pyrimidine nucleotides such as cCMP or cUMP could not be detected in *C. elegans* (data not shown).

### HPLC-MS/MS Quantitation of cAMP and cGMP in cGMP Signaling Mutants

**Figure 3:**
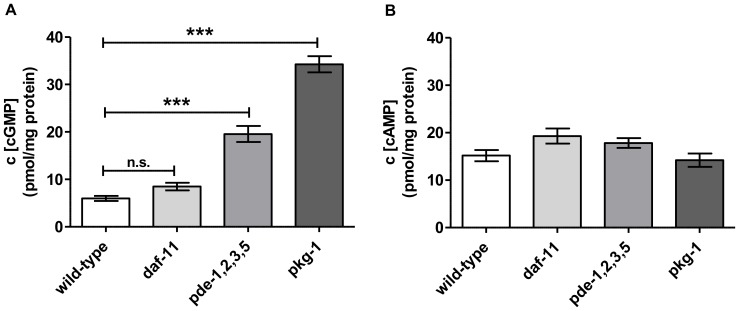
HPLC-MS/MS analysis of basal cGMP (A) and cAMP (B) concentrations in *C. elegans* wild-type (N2) and cGMP signaling mutants. Animals were grown to adult stage at 22°C on NGM plates and thus processed for HPLC-MS/MS cNMP quantitation. Data shown are the means ± SEM of seven independent experiments. P-values were calculated by means of one-way ANOVA with Bonferroni’s multiple comparison test with ***: p-value≤0.001, n.s.: not significant.

**Figure 4:**
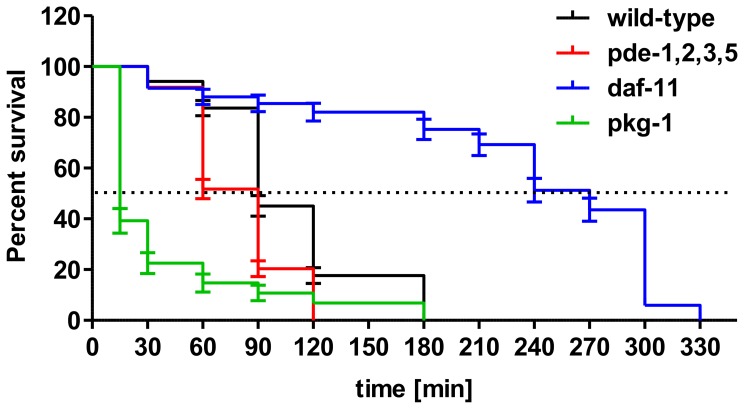
*C. elegans* stress response assay at 22°C in the presence of 10 mM H_2_O_2_. Animals were synchronized by picking L1 larvae, grown on NGM plates. At the stage of adulthood the animals were transferred to a 96-well plate containing 50 µl of 10 mM H_2_O_2_. Values represent means ± SEM of three independent experiments with wild-type = 153 animals, *pde-1,2,3,5* = 172 animals, *pkg-1* = 102 animals and *daf-11* = 117 animals. Survival rates were calculated *via* the Meier-Kaplan survival estimator and survival distributions were compared by Log-rank test. P-values were calculated by Log-rank test with p-values≤0.05 considered as significant. P-values describe the significant difference between wild-type and mutant strain survival during the whole time course; p-values for all mutants (*pde-1,2,3,5, pkg-1 and daf-11*): p<0.001. Dotted line: mean survival.

We next used our HPLC-MS/MS analysis system to measure cGMP concentrations in *C. elegans* mutants with altered levels of tolerance for oxidative (H_2_O_2_ -induced) stress. We further investigated the role of the guanylyl cyclase *daf-11* and *egl-4/pkg-1*, a gene encoding the cGMP-dependent protein kinase, in H_2_O_2_ -induced stress responses. Basal levels of cAMP and cGMP were determined in adult animals reared under standard *C. elegans* culture conditions. No significant change in cAMP concentrations was observed in any of the mutant strains compared to wild-type (Figure 3 B). Since the mutated genes affect the cGMP signaling pathway, this result for cAMP was expected. Interestingly, cGMP concentrations in the *daf-11* mutant also did not significantly differ from the levels observed in wild-type; however, previous data led us to expect a decrease in cGMP in *daf-11* mutants (Figure 3 A) [16]. The *pde-1,2,3,5* loss of function mutant, of which PDE-1, 3 and 5 are suggested to degrade cGMP, had a 4-fold higher concentration of cGMP than wild-type animals, confirming the data of Figure 1. Surprisingly, the *egl-4/pkg-1* mutant, having a mutation that causes complete deletion of the protein kinase G, showed even higher basal cGMP concentrations than the *pde-1,2,3,5* -quadruple mutant. The quadruple mutant harbored a 3.4-fold increase in cGMP compared to wild-type levels (Figure 3 A). In summary, the descending order of basal cGMP levels in all strains was *egl-4/pkg-1*>>*pde-1,2,3,5*>> *daf-11* =  wild-type. Interestingly, both mutants that showed the highest cGMP increase under H_2_O_2_ conditions also occurred to have the most severe phenotypes in lifespan decrease (see below, Figure 4, Table S2).

### Stress Tolerance Assays and cAMP and cGMP Quantitation in cGMP Signaling Mutants

Since *daf-11* and cGMP levels have been implicated in longevity and oxidative stress resistance, we analyzed the cGMP signaling mutants in the context of stress. Under 10 mM H_2_O_2_ conditions all the mutants displayed differences in tolerance to oxidative stress in comparison to wild-type animals reared under normal growth conditions (compare Figure 4 and Figure S2). The *egl-4/pkg-1* mutant strain showed the least tolerance to oxidative stress, as animals quickly died at this concentration of H_2_O_2_. After 15 min, 60% of the *egl-4/pkg-1* animals were already dead, leading to a median survival of only 15 min compared to 90 min of wild-type *C. elegans* (Figure 4). The *pde-1,2,3,5* quadruple mutant strain had the same median survival as wild-type, 90 min, but after 60 min almost 50% of the *pde-1,2,3,5* mutant animals were dead in comparison to 17% for wild-type. While the wild-type animals lived 180 min, the longest lifespan of *pde-1,2,3,5* quadruple mutants was only 120 min (Figure 4). Since the *daf-11* mutant strain had already been reported to have an increased tolerance for paraquat [16], an oxidative agent and one of the most widely used herbicides in the world, we also examined the influence of the stronger oxidative agent H_2_O_2_ on *daf-11* mutants. The *daf-11* mutant animals displayed a significant increase in lifespan in comparison to wild-type animals when exposed to H_2_O_2_. The mean survival of the *daf-11* mutants was 270 min, in contrast to 90 min for the wild-type strain (Figure 4). The oxidative stress resistance of the strains was as follows: *daf-11*>> wild-type >*pde-1,2,3,5*>> *egl-4/pkg-1* (Table S2).

**Figure 5:**
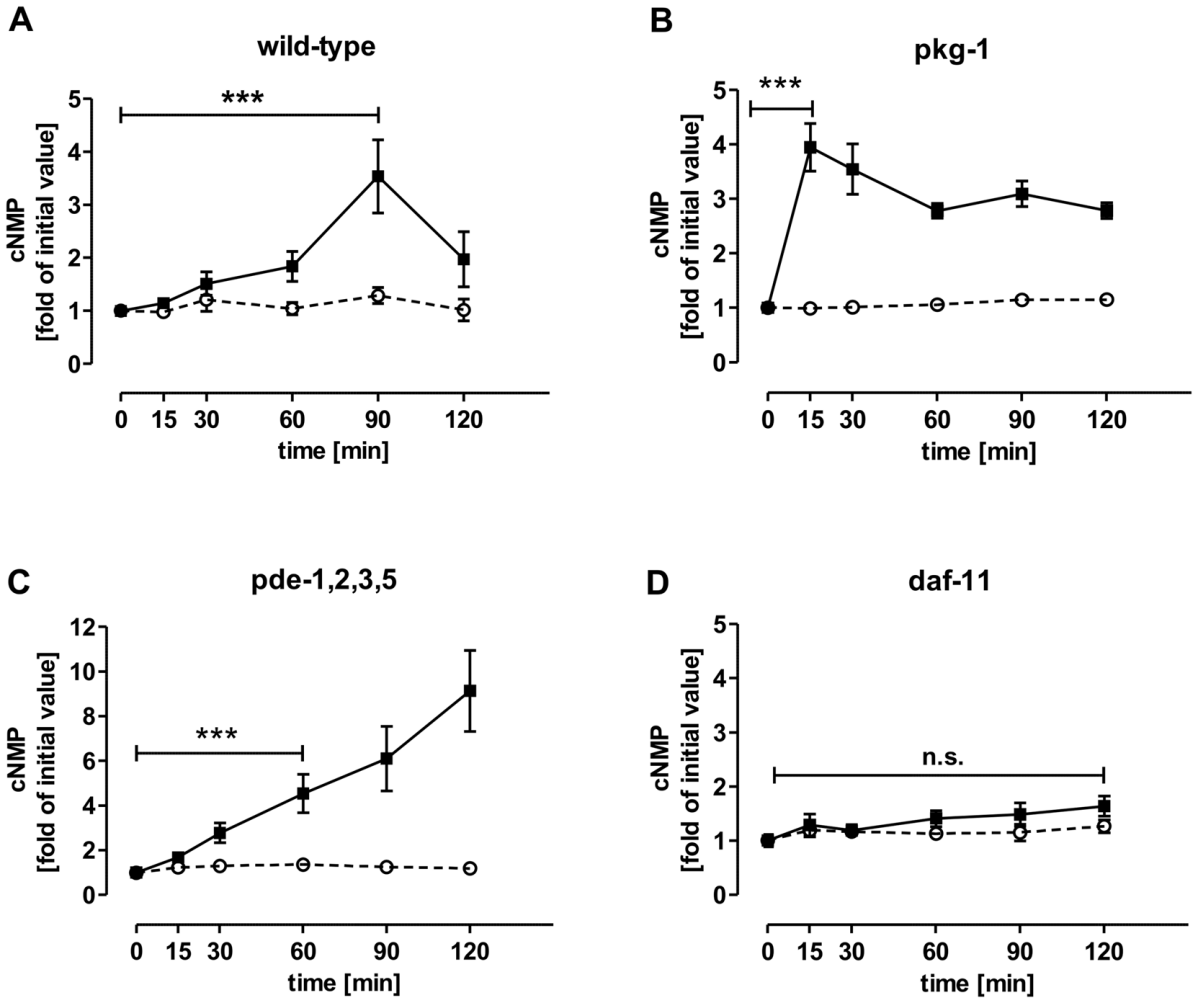
HPLC-MS/MS analysis of cGMP and cAMP of *C. elegans* mutants in the presence of H_2_O_2_. 72 h old, adult animals grown under normal conditions, were exposed to 10 mM of H_2_O_2_ and incubated for defined times at 22°C. After incubation, the animals were washed, extracted and and processed for HPLC-MS/MS quantification. **A** wild-type, **B** pkg-1 loss-of-function mutant, **C** quadruple pde loss-of-function mutant, **D** daf-11 (GC) loss-of-function mutant. Values normalized on the untreated control represent means ± SEM of at least three independent experiments. Please note the different scales of the y-axes of panel **C.** P-values were calculated by means of one-way ANOVA with Bonferroni’s multiple comparison test with ***: p-value≤0.001, n.s.: not significant. Please note in panel B and C: all times were tested for significant differences, but only the first incubation time with significance is highlighted with asterics. For all the following incubation times, the cGMP concentrations were also significantly higher (***:p-value≤0.001) than the untreated control (0 min) determined by one-way ANOVA with Bonferroni’s multiple comparison test. -**○**-: cAMP, -▪- : cGMP.

In order to examine the cAMP and cGMP concentrations in animals exposed to H_2_O_2_, the animals were synchronized, grown to adults and incubated with a final concentration of 10 mM H_2_O_2_. After incubation for 15, 30, 60, 90 and 120 min, the animals were processed for HPLC-MS/MS analysis. The wild-type strain showed a slow increase in cGMP that peaked after 90 min (Figure 5 A). Under the H_2_O_2_ conditions, the cGMP concentration of the *pkg-1* mutant strain increased in the first 15 min which was the earliest response of all strains tested and cGMP remained on a 3-fold higher level than the untreated control for the duration of the experiment (Figure 5 B). The cGMP level of the *pde-1,2,3,5*-quadruple mutant increased continuously throughout the 120 min duration of the experiment, and the strain accumulated the highest level of cGMP of all strains studied (Figure 5 C), in terms of the fold-change as well as the absolute amount (Table 5). Remarkably, *daf-11* mutants exposed to H_2_O_2_ did not display a significant change in cGMP concentration (Figure 5D) leading to the assumption that *daf-11* may be involved in mediating the H_2_O_2_-induced cGMP increase. However, while cGMP was regulated in different patterns in all mutant and wild-type strains (Figure 5), the cAMP levels in all strains remained unchanged. Absolute cGMP values in (pmol/mg protein) of all strains are listed in Table 5. The descending order of the cGMP level in the presence of H_2_O_2_ of all strains used in the assay is *pde-1,2,3,5*> *egl-4/pkg-1*>> wild-type>*daf-11* (Table 5).

**Table 5:** cGMP concentration of all *C. elegans* strains in the presence of 10 mM H2O2.

*Incubation time 10 mM* *H_2_* *O_2_*	*N2 c[cGMP] (pmol/mg* *protein) ± SEM*	*daf-11 c[cGMP] (pmol/mg* *protein) ± SEM*	*pkg-1 c[cGMP] (pmol/mg* *protein) ± SEM*	*pde-1,2,3,5 c[cGMP] (pmol/* *mg protein) ± SEM*
0 min	**4.7±0.86**	**8.3±0.93**	**34.7±2.5**	**17.3±2.2**
15 min	4.4±0.49	10.7±1.7	135±12.8***	29.3±3.0
30 min	5.3±0.58	9.9±0.90	120±13.3***	45.8±5.6**
60 min	7.4±1.01	11.7±1.2	97.4±4.9***	79.2±8.8***
90 min	13.8±2.9***	12.3±1.8	108±6.2***	104±14.5***
120 min	6.2±1.0	13.6±1.6	98.7±4.6***	155±12.0***

Animals were exposed to 10 mM H_2_O_2_ and incubated for defined times at 22°C. After defined incubation times animals were processed for HPLC-MS/MS analysis. Data shown are means ± SEM of three to four independent experiments in pmol/mg. P-values were calculated by means of one-way ANOVA with Bonferroni’s multiple comparison test with ***: p-values <0.001, significance was calculated of values compared to initial untreated values, n.s.: not significant.

### qRT-PCR Analysis of cGMP Signaling Mutants

**Figure 6:**
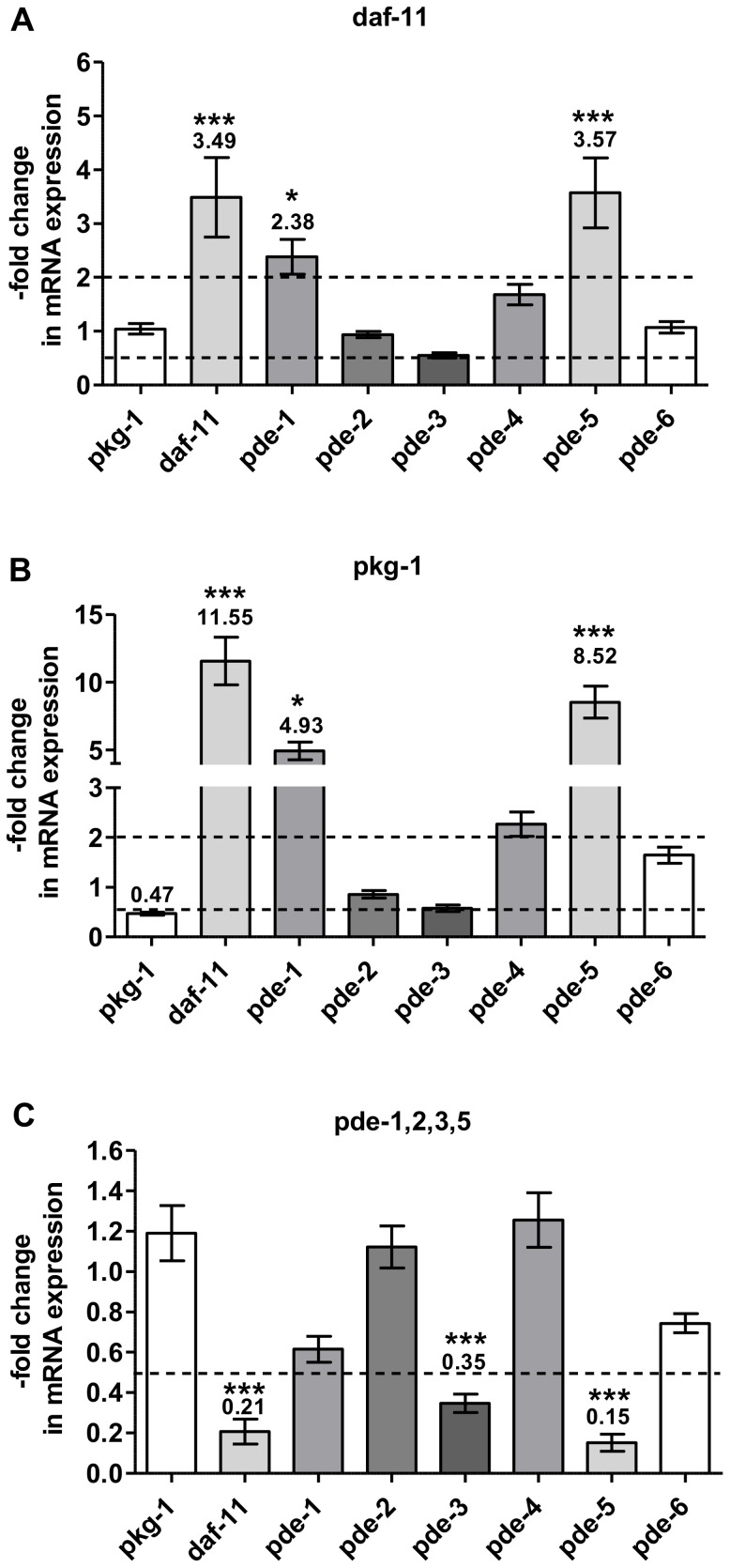
mRNA expression of *C. elegans* pde genes, daf-11 gene and pkg-1 gene on the basis of qRT-PCR analysis. Animals were grown to adult stage (72 h) under normal conditions on NGM plates at 22°C. 500–1000 worms were harvested and processed for RNA extraction by freeze-crack and thus qRT-PCR analysis was conducted in order to determine background gene regulation in the cGMP-signaling pathway. Gene expression of **A** the daf-11, **B** the pkg-1 and **C** the pde-quadruple mutant background. Data shown are means ± SEM of 3 to 8 independent experiments normalized on wild-type level. Data have been normalized to the geometric mean of two housekeeping genes Y45F10D.4 and pmp3 chosen. P-values were calculated by means of one-way ANOVA with Bonferroni’s multiple comparison test with ***: p-value≤0.001; *: p-value≤0.05. Please note the different scales of panel A–C. Dashed lines depict 2-fold increase or decrease in mRNA expression compared to wild-type.

Biosynthesis and degradation of cNMPs occurs in the context of a wide network of interconnected cyclases and phosphodiesterases is subject to allosteric influences and feedback regulation. We considered that feedback regulation might also include regulation at the transcriptional or mRNA stability level. We therefore analyzed the relative mRNA abundance of the *pde* gene class as well as mRNA levels of *egl-4/pkg-1* and *daf-11* in the cGMP signaling mutants studied above. Wild-type, *daf-11, pkg-1* and *pde-1,2,3,5* adult animals were harvested and processed for RNA preparation. The levels of *daf-11, pkg-1, pde-1, pde-2, pde-3, pde-4, pde-5* and *pde-6* gene expression were then quantified by qRT-PCR for each of the strains. Although the reported mutation of the *daf-11* strain leading to an entire deletion of the cyclase domain of *daf-11* on the protein level, mRNA levels of *daf-11* were up-regulated (about 3.5-fold) [29]. Interestingly, the gene expression levels for *pde-1* and *pde-5*, encoding enzymes postulated to degrade cGMP [16], [18], were also elevated in *daf-11* mutants (2.4-fold and 3.6-fold, respectively) (Figure 6 A). The *egl-4/pkg-1* mutant, harboring a nonsense mutation, showed a reduction of *egl-4/pkg-1* on the mRNA level (0.47-fold), and a significant elevation of *daf-11* gene expression (11.6-fold), and an increase in *pde-1* and *pde-5* mRNA expression as well (Figure 6 B). The mRNA accumulation profiles for *daf-11* and *egl-4/pkg-1* mutants are remarkably similar (Figure 6). By contrast, quantitation of the mRNA levels of the *pde-1,2,3,5*-quadruple mutant revealed that *daf-11* gene expression was strongly reduced (0.21-fold) in the *pde-1,2,3,5*-quadruple mutant (Figure 6 C). In all mutants tested, the regulation of the *pde-4* gene, which is a phosphodiesterase with a predicted preference for cAMP degradation, was not significantly affected; nor were effects noted for the expression of *pde-2* or *pde-6*, whose cNMP specificities have not been described yet (Figure 6, Table S3).

## Discussion

### cAMP and cGMP *in vivo* Analysis of *C. elegans via* HPLC-MS/MS

Previous studies using *C. elegans* phosphodiesterase and adenylyl or guanylyl cyclase mutants postulated decreased or enhanced levels of cAMP or cGMP without biochemically analyzing the actual cAMP or cGMP content of the animals. The assumptions of cNMP levels were based on the nature of the enzyme activity that was defective in the corresponding mutant or by exposing animals to high concentrations of exogenous cNMPs [16], [18], [19]. These assumptions may be incorrect, as the pathways that lead to production and degradation of cAMP and cGMP are interconnected and extensively cross-regulated. A defect in one element of this network could lead to unexpected consequences with respect to the balance of cNMPs in the cell.

We developed a HPLC-MS/MS methodology combined with a particular preparation of *C. elegans* that is suitable for the specific and sensitive simultaneous detection of cAMP and cGMP in the nematode. For validating the HPLC-MS/MS methodology for *C. elegans* oxidative stress assays, we examined phosphodiesterase mutants, we assayed animals defective in particular adenylyl or guanylyl cyclases, and we used forskolin to activate adenylyl cyclases in *C. elegans*. Indeed, for the first time, we report definitive data that will allow for direct comparisons of cNMP levels in whole animals. In our initial proof-of-principal experiments, we observed higher concentrations of cAMP in *pde-4* loss-of-function mutants in comparison to wild-type; whereas cGMP levels were not altered. Additionally, a mutant containing loss of function mutations in the four phosphodiesterase genes *pde-1*, *pde-2, pde-3* and *pde-5* showed a significant elevation in cGMP levels (PDE-1 and PDE-5 were postulated to degrade cGMP [16]) (Figure 1). By analyzing the influence of forskolin, a direct activator of adenylyl cyclases, we observed an enhanced accumulation of cAMP in wild-type *C. elegans*. These initial results have allowed us to validate our HPLC-MS/MS methodology in the *C. elegans* system for direct comparisons between different cNMP levels.

Our data confirmed previous assumptions that cAMP levels are upregulated in *pde-4* mutants (Figure 1), which are defective for a phosphodiesterase postulated to degrade cAMP. Both *pde-4* loss –of-function and *acy-1(gf)* gain-of-function mutants display increased axon-regrowth behavior, and both strains are predicted to harbor elevated levels of cAMP [19]. By contrast, we detected no increase of cAMP in the same *acy-1(gf)* strain (Figure S1). These unexpectedly contradictory results argue for a more complex role for cNMPs in this interesting biological process. Similarly, our data also support other assumptions that cGMP levels are increased in a *pde-1,2,3,*5 quadruple mutant that displays enhanced photocurrent [18] (Figure 1). With our new MS technology experiments designed to uncover additional roles for cNMPs in axon-regeneration and phototransduction can be performed with confidence. We have used similar methodologies in analyses of cNMP levels in mammalian cell lines, studies which allowed for the definitive demonstration of the presence of the pyrimidine nucleotides cCMP and cUMP [30]. cCMP or cUMP could not be detected in *C. elegans,* a finding which not only highlights the specificity of our technology, but also points to an evolutionary and species-specific pattern of cNMP metabolism.

### Analysis of cGMP Signaling Mutants and their Roles in the H_2_O_2_ Stress Response

#### Correlation of cGMP concentrations and lifespan

*C. elegans* is a useful organism for aging and oxidative stress research [31], [32], [33], [34]. As for mammals, restricted caloric up-take or moderate oxidative stress can extend the lifespan of *C. elegans.* By contrast, high levels of oxidative stress provoke cell death *via* protein, lipid or DNA damage in both mammals and *C. elegans* [35], [36], [37]. Long-lived mutants of *C. elegans,* such as *daf-2, age-1,* and *sir-2.1,* express higher levels of the detoxifying enzymes catalase and superoxide dismutase, allowing for increased oxidative stress resistance, a phenotype that is correlated with lifespan extension [16], [32], [33], [38], [39]. Mutants, lacking DAF-11, a receptor-bound guanylyl cyclase, also live about 30% longer than wild-type animals and are more resistant to paraquat, an oxidative agent used as herbicide [16]. From these and other data, one might hypothesize that low cGMP levels provoke lifespan extension and mediate resistance to oxidative stress. Since we are now able to quantify cAMP and cGMP in *C. elegans via* HPLC-MS/MS, we utilized this new methodology to test this hypothesis by directly measuring cNMP levels in *daf-11* and related mutants after exposure to 10 mM H_2_O_2._

In order to reveal correlations between cNMP levels and stress response pathways, we analyzed loss-of-function *C. elegans* mutants of the cGMP metabolic pathway. In both wild-type and the quadruple phosphodiesterase *pde-1,2,3,5* mutant, the levels of cGMP increased progressively in response to the time of exposure to H_2_O_2_, with the highest levels of cGMP accumulating in the quadruple mutant. The *pde-1,2,3,*5 mutants also have higher than normal levels of cGMP when untreated, while *daf-*11 mutants do not (Figure 3). In contrast, the pkg-1 strain had the earliest strongest cGMP increase, already after 15 min H_2_O_2_ incubation. These two described mutant strains also displayed the greatest sensitivity to oxidative stress (Figures 4 and 5). Surprisingly, basal cGMP levels in guanylyl cylclase *daf-11* mutants were unexpectedly unchanged in comparison to wild-type levels (Figure 5). Thus, while the *pde-1,2,3,5 and pkg-1 *data support the hypothesis that cGMP levels are inversely correlated with oxidative stress resistance, the *daf-11* data do not. Remarkably, cAMP concentrations were not affected by oxidative stress in any of the strains we tested, which points to a particular role for cGMP in oxidative stress mechanisms of *C. elegans* (Figure 5).

Strikingly, the cGMP levels of the untreated *egl-4*/*pkg-1* mutants were even higher than those of the *pde-1,2,3,5* mutant (Figure 5). *Egl-4/pkg-1* is one of two closely related cGMP-dependent protein kinase genes in *C. elegans.* The increased level of cGMP in *egl-4/pkg-*1 mutants, coupled with the cell’s inability to transduce signals through PKG-1, may provide indications of a compensatory feed-back mechanism in the cGMP signaling pathway. Such compensatory mechanisms have been reported for superoxide dismutase mutations in *C. elegans*. For example, in *sod-2* deletion mutants, *sod-1, sod-3* and *sod-4 *mRNA levels were significantly elevated [34].

Under normal growth conditions, we did not observe an extended lifespan for *pkg-1* or *daf-11* single mutants or the *pde-1,2,3,5* quadruple mutant (Figure S2). These findings are contrary to lifespan data for *pkg-1* and *daf-11* mutants published by Hahm *et al.* 2009 and Hirose *et al.* 2003, even though our experimental approach to measuring lifespan was similar to that of Hahm *et al.*, in that we utilized FUDR to prevent the development of progeny. However, we utilized FUDR at 0.05 mg/ml while Hahm *et al*. used a higher concentration (0.1 mg/ml). Differences in lifespan may also be attributable to the growth temperature; our strains were reared at 22°C, while the Hahm experiments were performed at 20°C. Similarly the lifespan measurements performed for *egl-4/pkg-1* by Hirose et al. were performed at 20°C; additionally, Hirose *et al.* assessed a different set of alleles. (The *pkg-1* mutants did display the typical enlarged body size phenotype [40], [41].).

Nevertheless, our observation, coupled with the fact that *egl-4/pkg-1* and the *pde*-1,2,3,5 quadruple mutants have high basal cGMP contents (Figure 3), support a hypothesis that cGMP is not the main player in enhanced or decreased lifespan under normal growth conditions. Thus cGMP has differing influences on *C. elegans*’ lifespan depending on the environmental conditions. It should be noted that our data obtained with worms cannot necessarily be transferred to mammals.

#### The impact of background gene expression on the mutant cGMP concentrations

**Figure 7:**
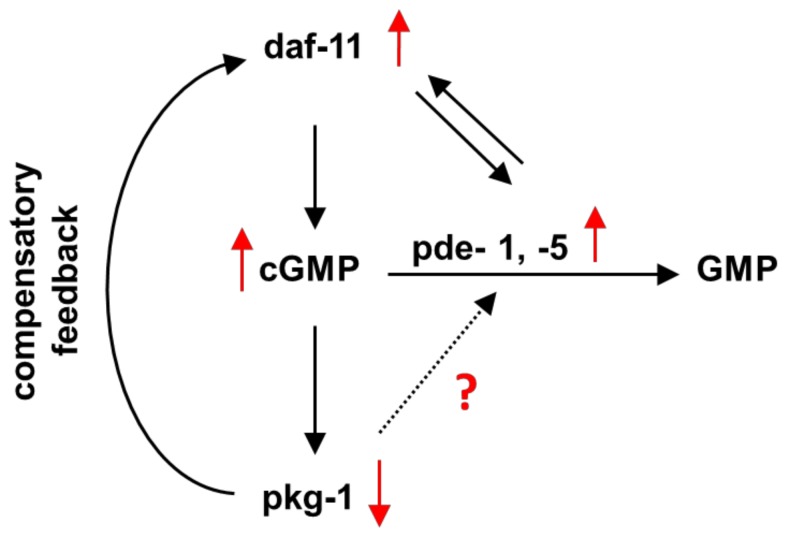
Crosstalk of daf-11/pkg-1/pde signaling in *C. elegans* analyzed by qRT-PCR and HPLC-MS/MS analysis of cGMP. Analysis of normal grown, adult cGMP signaling mutants revealed a compensatory feedback response in *egl-4/pkg-1* deletion mutants towards *daf-11*, with enhanced *daf-11* gene expression, resulting in high cGMP concentrations and thus enhanced *pde-1* and *pde-5* gene expression. *Vice versa*, pde- deletion mutants showed reduced *pde-1* and *pde-5* gene expression connected with decreased daf-11 expression levels pointing to a direct dependency of *daf-11* and *pde-1* and *pde-5* gene expression in both directions. Since *egl-4/pkg-1* gene expression was not affected in the *daf-11* loss of function or the pde-deletion background, there is only a feedback regulation based on *egl-4/pkg-1* gene expression. Red arrows stand either for down- (↓) or up-regulation (↑) of genes.

As mentioned above, our data provide evidence for compensatory feedback mechanisms that might explain the high basal cGMP levels we observed in *egl-4/pkg-1* mutants (Figure 3). We investigated effects on mRNA accumulation/transcription using qRT-PCR analysis of untreated mutants defective in cGMP signaling or metabolic pathways. Indeed, in *egl-4/pkg-1* mutants, the *daf-11* guanylyl cyclase mRNA was strongly up-regulated (11.6-fold) in comparison to the level observed in wild-type (Figure 6). This may lead to increased DAF-11 enzyme production, and hence, the high basal cGMP levels (Figure 7). The immediate and significant increase in cGMP levels observed in *egl-4/pkg-1* mutants in the presence of H_2_O_2_ (Figure 5) likely reflects the overabundance of *daf-11* mRNA, underlining the mediator function of this guanylyl cyclase in the oxidative stress response.

The increase in *daf-*11 mRNA, and accompanying high cGMP levels, detected in *egl-4/pkg-1* mutants may also be responsible for the up-regulation of *pde-1* and *pde-5* gene expression we observed (Figure 7). Similar regulation of enzymes with opposite activities with respect to cGMP metabolism would impart an ability of the cell to maintain cGMP homeostasis; although, in *egl-4/pkg-1* mutants, perfect homeostasis is not achieved (Figure 3). If this model is correct, we can predict that increases in cGMP might provoke an increase in *pde-1* and *pde-5* gene expression, and decreases in cGMP would lead to upregulation of *daf-11*. Indeed, we do observe a decrease in *daf-11* mRNA levels in the *pde-1,2,3,5* quadruple mutant (Figure 7). The pde-1,2,3,5 quadruple phosphodiesterase mutant has drastically elevated cGMP levels, and not surprisingly, the expression level of the *daf-11* guanylyl cyclase is reduced. One might conclude that the reduced level of *daf-11* mRNA in this genetic background is not compromised, as the cGMP product is more stable (Figure 3). These results confirm the assumption that cNMP-degrading enzymes have a stronger impact on intracellular cNMP levels in *C. elegans* than cNMP-generating enzymes. Taken together, the collective data highlight the potential for cross-talk between cGMP generating and degrading enzymes in *C. elegans* (Figure 7).

*daf-11* mRNA is down-regulated in the *pde-1,2,3,5* quadruple mutant (Figure 6), yet the cGMP concentration increases in this mutant in the presence of H_2_O_2_ (Figure 5). In contrast to mammals, whose soluble guanylyl cyclases are activated by NO [42], [43], soluble guanylyl cyclases of *C. elegans* are activated by oxygen (O_2_) (Figure S4) [44]. Therefore, it is possible that in the presence of H_2_O_2_, detoxifying enzymes such as catalases, which convert H_2_O_2_ into H_2_O and O_2_, might indirectly activate sGCs and thus contribute to cGMP formation.

An ability to synthesize cGMP independently of DAF-11 can explain why *daf-11* mutants harbor normal cGMP levels (Figure 3). This is remarkable when considering that *daf-11* mutants also have moderately increased levels of the phosphodiesterases *pde-5* and *pde-1* (Figure 6). These data confirm the results of the *egl-4/pkg-1* and *pde-1,2,3,5* quadruple mutant indicating a strong correlation between *daf-11* and *pde-5* gene expression (Figure 7). When *daf-11* is upregulated *pde-5* is elevated and when *pde-5* is strongly down-regulated, *daf-11* gene expression is also reduced (Figure 6 C).

### Limitations of our Study

Compartmentalization and short local increases in cNMPs could be taken into consideration for explaining the effects of the mutations in cNMP pathways. For example, aberrant compartmentalization in *acy-1(gf)* mutants may contribute to axon regeneration and such subtle cellular displacements might be below our ability to detect in our assays [45]. Also, our MS-based method cannot analyze cNMP levels in specific cellular compartments. Furthermore, our studies underline that only relying on mutations is not sufficient for correct interpretation of results, since the mutations do not necessarily correlate with the situation of intracellular cNMP concentrations *in vivo* (Figure S1).

We identified the receptor-bound guanylyl cyclase DAF-11 as the mediator of H_2_O_2_-induced cGMP increase in *C. elegans* and found that the cGMP concentration inversely correlates with the life expectancy of the nematode in the presence of H_2_O_2_ while the cAMP pathway does not seem to be part of the oxidative stress response. This was not only confirmed by HPLC-MS/MS quantitation but also by qRT-PCR analysis, which revealed no regulation of the *pde-4* gene, encoding for a cAMP degrading PDE, in any cGMP signaling mutant (Figure 6). Our data provides evidence that DAF-11 might not be the only guanylyl cyclase that generates cGMP in response to H_2_O_2_. It is likely that soluble guanylyl cyclases are also involved in this process.

Our analysis of the gene expression profile in *C. elegans* cGMP-signaling mutants *via* RT-PCR assays showed that it is important to take into consideration that *daf-11/pkg-1/pde* crosstalk occurs. In contrast to *C. elegans*, oxidative stress studies with HEK293 cells overexpressing the DAF-11 ortholog pGC-A [46], showed a simultaneous regulation of cAMP and cGMP (Figure S3) indicating that cross-talk of cGMP and cAMP occurs in mammalian cells upon H_2_O_2_ exposure. While these results highlight the differences between the two systems, *C. elegans* is a useful model organism for initial exploration experiments in cNMP signaling upon oxidative stress, because the reduced level of cross-talk and absence of NO responses might allow for a more complete elucidation of novel cNMP pathways [47], [48].

The particular interpretation of the *C. elegans* cGMP regulation under normal and oxidative stress conditions could only be realized by profiling the levels of mRNAs of other components in the cGMP signaling pathway in order to comprehend the impacts of specific gene function and environmental stressors on the biochemical results. Thus, it is important not only to analyze *C. elegans* mutants by a biochemical or a genetic approach but by the combination of both.

### Future Perspectives

Due to the lack of intense immunochemistry in *C. elegans* in the past, the field of protein biochemistry is just evolving providing reliable antibodies in order to study the effects of H_2_O_2_ on the protein level. Therefore, on the one hand future studies will focus on elucidating the impact of oxidative stress on the protein level of *daf-11*, *egl-4/pkg-1* and *pdes*. On the other hand, the pharmacological manipulation of *C. elegans* using guanylyl cyclase activators/inhibitors, PDE inhibitors or membrane permeable cNMP analogs, with and without exposition to oxidative stress will provide further information in how far the members of the cGMP-signaling pathway are involved in the oxidative stress response.

## Supporting Information

Figure S1 **HPLC-MS/MS quantitation of cAMP (A) and cGMP (B) of wild-type (N2) and** ***acy-1*** **gain-of-function animals.** Nematodes were synchronized and grown under normal conditions on NGM agar plates to adulthood at 22°C. No significant difference in **A** cAMP and **B** cGMP concentrations could be detected. Values represent means ± SEM normalized on wild-type cNMP concentrations, n.s.: not significant.
(TIF)

Figure S2 **Lifespan analysis of** ***C. elegans*** **at 22°C under normal growth conditions.** NGM plates were supplemented with 0.05 mg/ml FUDR in order to inhibit progeny overgrowth. Data shown are means ± SEM of three independent experiments with N2 = 267 animals, **A** *pde-1,2,3,5* = 259 animals, **B** *pkg-1* = 277 animals and **C** *daf-11* = 281 animals. Survival rates were calculated *via* the Meier-Kaplan survival estimator and survival distributions were compared by logrank test. P-values were calculated by Log-rank test with p-values≤0.05 considered as significant. P-values depicted in panels **A–D** describe the significant difference between wild-type and mutant strain survival during the whole time course. Dotted line: mean survival.
(TIF)

Figure S3 **HPLC-MS/MS analysis of Hek293 WT vs. Hek293 overexpressing pGC-A in the presence of H_2_O_2_.** Panels **A**, **C**, **E**, **G** display cGMP and panels **B**, **D**, **F**, **H** cAMP levels normalized on the cNMP values of the untreated control. Values represent means ± SEM of three independent experiments. P-values were calculated by means of ANOVA Bonferroni’s multiple comparison test with ***: p-value≤0.001, **: p-value≤0.01, *: p-value≤0.05, n.s.: not significant. Please note the different scale of the y-axes of panel **A**.
(TIF)

Figure S4 ***C. elegans*** **soluble guanylyl cyclase dependent cGMP signaling in ciliated neurons.** Soluble guanylyl cyclases (sGC) GCY-35 and GCY-36 of *C. elegans* are activated by O_2_. Subsequently, GTP is converted to cGMP. cGMP effectors are cGMP-gated channels (TAX-2 and TAX-4), PKG-1 (protein kinase 1) and most likely PDE 1, 2 and 5 (cGMP degrading phosphodiesterases). Activation of sGC results in avoidance of hyperoxia, bordering and aggregation of the animals on a bacterial lawn [46].
(TIF)

Figure S5 **Concentration-response curve of wild-type** ***C. elegans*** **incubated with H_2_O_2_.** 72 h old adult animals were incubated with increasing concentrations of H_2_O_2_∶2.5 mM, 5 mM, 7.5 mM, 10 mM and 15 mM. After 2 h, the living animals were identified by tapping the nematodes with a plantinum wire-pick. The animals were counted as alive when they responded to the pick, otherwise they were counted as dead. With a concentration of 15 mM, almost all animals were dead after 2 h of incubation. Therefore, a final concentration of 10 mM H_2_O_2_ was chosen, in order to conduct a time-dependent H_2_O_2_ stress assay.
(TIF)

Table S1 ***C. elegans*** **cGMP signaling pathway mutants used in this study.**
(DOCX)

Table S2 ***C. elegans*** **survival in the presence of 10 mM H2O2 (up to 270 min).**
(DOCX)

Table S3 **qRT-PCR analysis of** ***C. elegans*** **wild-type and mutants.**
(DOCX)
