# Correction: The Receptor-Bound Guanylyl Cyclase DAF-11 Is the Mediator of Hydrogen Peroxide-Induced cGMP Increase in *Caenorhabditis elegans*

**DOI:** 10.1371/annotation/e9f85804-f0d5-41c8-8b0b-afcd9fe69fd6

**Published:** 2013-11-11

**Authors:** Ulrike Beckert, Wen Yih Aw, Heike Burhenne, Lisa Försterling, Volkhard Kaever, Lisa Timmons, Roland Seifert

An error was introduced in the preparation of this article for publication.

The correct title should read: The Receptor-Bound Guanylyl Cyclase DAF-11 Is the Mediator of Hydrogen Peroxide-Induced cGMP Increase in *Caenorhabditis elegans*

The correct citation is: Beckert U, Aw WY, Burhenne H, Försterling L, Kaever V, et al. (2013) The Receptor-Bound Guanylyl Cyclase DAF-11 Is the Mediator of Hydrogen Peroxide-Induced cGMP Increase in *Caenorhabditis elegans*. PLoS ONE 8(8): e72569. doi:10.1371/journal.pone.0072569 

