# Supplementary figures and images for: The Receptor-Bound Guanylyl Cyclase DAF-11 Is the Mediator of Hydrogen Peroxide-Induced Cgmp Increase in Caenorhabditis elegans
Source: PLoS One. 2013 Aug 27;8(8):e72569. doi: 10.1371/journal.pone.0072569 (PMC3754915; doi:10.1371/journal.pone.0072569)

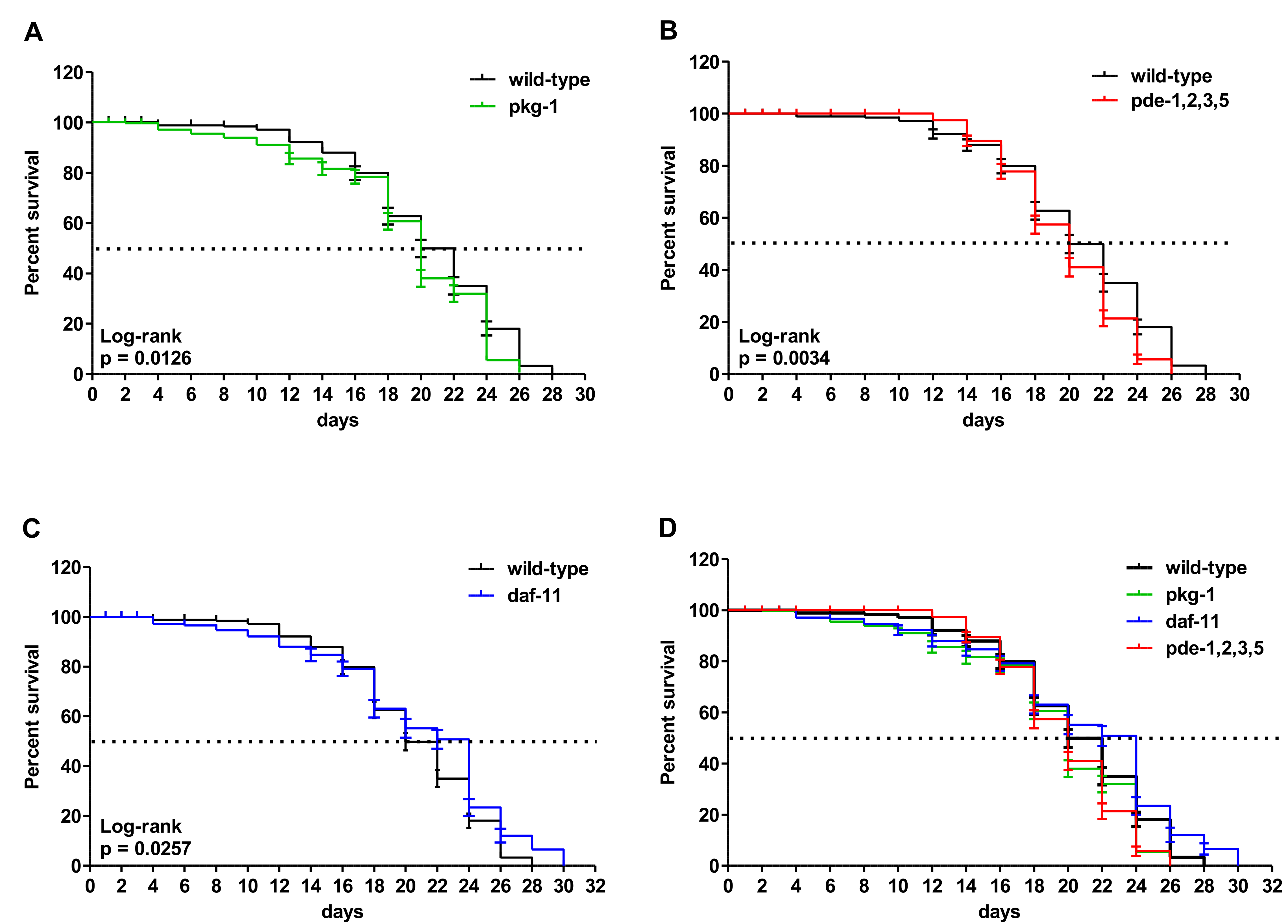

Supplement: Figure S2 — Lifespan analysis of C. elegans at 22°C under normal growth conditions. NGM plates were supplemented with 0.05 mg/ml FUDR in order to inhibit progeny overgrowth. Data shown are means ± SEM of three independent experiments with N2 = 267 animals, A pde-1,2,3,5 = 259 animals, B pkg-1 = 277 animals and C daf-11 = 281 animals. Survival rates were calculated via the Meier-Kaplan survival estimator and survival distributions were compared by logrank test. P-values were calculated by Log-rank test with p-values≤0.05 considered as significant. P-values depicted in panels A–D describe the significant difference between wild-type and mutant strain survival during the whole time course. Dotted line: mean survival. (TIF) [file pone.0072569.s002.tif]

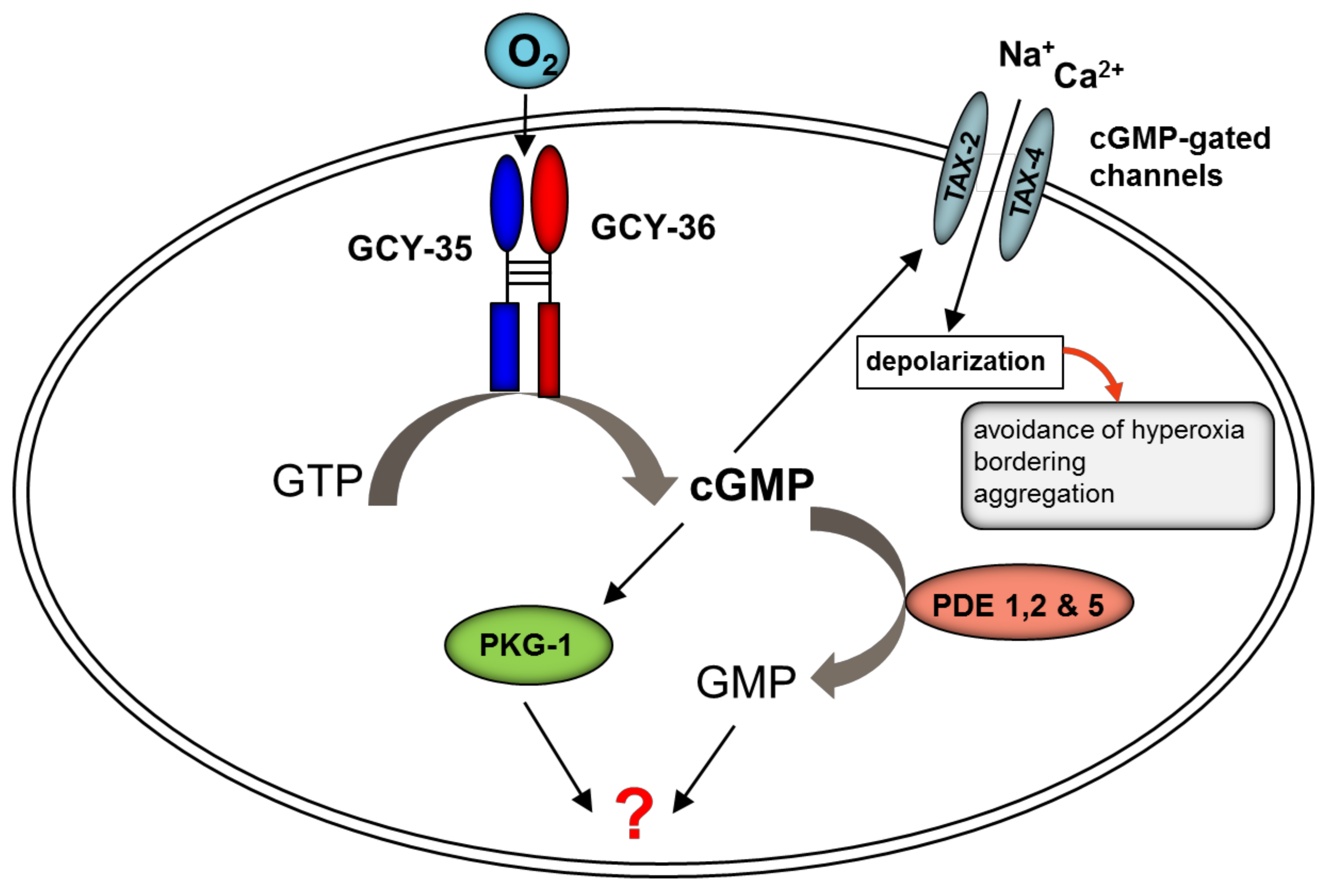

Supplement: Figure S4 — C. elegans soluble guanylyl cyclase dependent cGMP signaling in ciliated neurons. Soluble guanylyl cyclases (sGC) GCY-35 and GCY-36 of C. elegans are activated by O2. Subsequently, GTP is converted to cGMP. cGMP effectors are cGMP-gated channels (TAX-2 and TAX-4), PKG-1 (protein kinase 1) and most likely PDE 1, 2 and 5 (cGMP degrading phosphodiesterases). Activation of sGC results in avoidance of hyperoxia, bordering and aggregation of the animals on a bacterial lawn [46]. (TIF) [file pone.0072569.s004.tif]
