# Supplementary material for: The Receptor-Bound Guanylyl Cyclase DAF-11 Is the Mediator of Hydrogen Peroxide-Induced Cgmp Increase in Caenorhabditis elegans
Source: PLoS One. 2013 Aug 27;8(8):e72569. doi: 10.1371/journal.pone.0072569 (PMC3754915; doi:10.1371/journal.pone.0072569)
Supplement: Table S1 — C. elegans cGMP signaling pathway mutants used in this study. (DOCX) [file pone.0072569.s006.docx]

Supplemental Table S1: *C. elegans* cGMP signaling pathway mutants used in this study

| *strain* | *gene (allele)* | *molecular change* | *protein effects* | *resulting phenotype* |
| --- | --- | --- | --- | --- |
| N2 var. Bristol | wild-type | none | none | wild-type [1] |
| MT 1074 | pkg-1 (n479)IV | substitution | nonsense, R to opal stop (415) [www.wormbase.org] | aldicarb resistent butanone, diacetyl, NaCl & lysine chemotaxis defective [2] egglaying defective ,egg retention [2,3], dark intestine, long bodysize [2,3,4], neuron function reduced [5] |
| DR 47 | daf-11 (m47)V | molecular change in the predicted kinase domain | nonsense, entire deletion of GC domain [11] | CO_2_ avoidance [6], *dauer* constitutive [7,8], extended lifespan, sod-3 upregulation [9], pde-1 & pde-5 downregulation [9], paraquat resistent [9] photocurrent defective [10], reduced cGMP? [9] |
| TQ 1828 | pde-1(nj57),pde-5(nj49) I;pde-3(nj59) II;pde-2(tm3098)III | frame shift/ deletion in catalytic domaine | nonsense, disruption of catalytic domaine [10] | photocurrent potentiated [10] |

**Supplemental References**

1. Brenner, S. (1974) The genetics of *Caenorhabditis elegans*. Genetics 77, 71-94.
2. Daniels, S. A., Ailion, M., Thomas, J.H., Sengupta, P. (2000) Egl-4 acts through a transforming growth factor-b/SMAD pathway in *Caenorhabditis elegans* to regulate multiple neuronal circuits in response to sensory cues. Genetics 156, 123–141.
3. Trent, C., Tsuing, N., Horvitz, H. R. (1983) Egg-laying defective mutants of the nematode *Caenorhabditis elegans*. Genetics 104, 619–647
4. Hirose, T., Nakano, Y., Nagamatsu, Y., Misumi, T., Ohta, H., Ohshima, Y. (2003) Cyclic GMP-dependent protein kinase EGL-4 controls bodysize and lifespan in C. elegans. Development 130, 1089–1099.
5. L’Etoile, N. D., Coburn, C. M., Eastham, J., Kistler, A., Gallegos, G., Bargmann, C. I. (2002) The cyclic GMP-dependent protein kinase EGL-4 regulates olfactory adaptation in *C. elegans*. Neuron 36, 1079–1089.
6. Hallem, E. A., Sternberg, P. W. (2008) Acute carbon dioxide avoidance in *Caenorhabditis elegans*. Proc. Natl. Acad. Sci. USA 105, 8038-8043.
7. (104) Riddle, D. L., Albert, P. S., MacMorris, M. A. (1981) Interacting genes in nematode dauer larva formation. Nature 290, 668-71
8. Fierro-Gonzalez, J. C., Cornils, A., Alcedo, J., Miranda-Vizuete, A.,Swoboda, P. (2011) The thioredoxin TRX-1 modulates the function of the insulin-like neuropeptide DAF-28 during dauer formation *in Caenorhabditis elegans*. PLoS One 6, e16561.
9. Hahm, J. H., Kim, S., Paik, Y. K. (2009) Endogenous cGMP regulates adult longevity via the insulin signaling pathway in Caenorhabditis elegans. Aging Cell 8, 473-83.
10. Liu, J., Ward, A., Gao J.,, Dong, Y., Nishio, N., Inada, H., Kang, L. Yu, Y., Ma, D., Xu, T., Mori, I., Xi, Z., Xu, X. Z. S. (2010) C. elegans phototransduction requires a G protein-dependent cGMP pathway and a taste receptor homolog, Nat Neurosci. 13, 715–722.
11. Murakami M, Koga M, Oshima Y (2001) DAF-7/TGF-b expression required for the normal larval development in C. elegans is controlled by a presumed guanylyl cyclase DAF-11. Mechanisms of Development: 109: 27-35.
