# Supplementary material for: The Receptor-Bound Guanylyl Cyclase DAF-11 Is the Mediator of Hydrogen Peroxide-Induced Cgmp Increase in Caenorhabditis elegans
Source: PLoS One. 2013 Aug 27;8(8):e72569. doi: 10.1371/journal.pone.0072569 (PMC3754915; doi:10.1371/journal.pone.0072569)
Supplement: Table S2 — C. elegans survival in the presence of 10 mM H2O2 (up to 270 min). (DOCX) [file pone.0072569.s007.docx]

Supplemental Table S2: *C. elegans* survival in the presence of 10 mM H_2_O_2_ (up to 270 min).

| *incubation time 10 mM H_2_O_2_* | *N2 survival in % ± SEM* | *daf-11 survival in % ± SEM* | *pkg-1 survival in % ± SEM* | *pde-1,2,3,5 survival in % ± SEM* |
| --- | --- | --- | --- | --- |
| 0 min | 100 | 100 | 100 | 100 |
| 15 min | 100 | 100 | 39 ± 4.8 | 100 |
| 30 min | 94 ± 2 | 92 ± 2.6 | 23 ± 4.1 | 92 ± 2.1 |
| 60 min | 84 ± 3 | 88 ± 3 | 15 ± 3.5 | 52 ± 3.8 |
| 90 min | 45 ± 4 | 85 ± 3.3 | 11 ± 3.1 | 20 ± 3.1 |
| 120 min | 18 ± 3 | 82 ± 3.5 | 6.8 ± 2.5 | 1.7 ± 0.9 |
| 180 min | 0 | 75 ± 3.9 | 0 | 0 |
| 270 min | 0 | 44 ± 4.6 | 0 | 0 |

Animals were synchronized by picking L1 larvae, grown on NGM plates and at the stage of adulthood transferred to a 96-well plate containing 10 mM H_2_O_2_. Values represent means ± SEM of three independent experiments with N2= 153 animals, pde-1,2,3,5 = 172 animals, daf-11= 117 animals and pkg-1= 102 animals.
