# Supplementary material for: The Receptor-Bound Guanylyl Cyclase DAF-11 Is the Mediator of Hydrogen Peroxide-Induced Cgmp Increase in Caenorhabditis elegans
Source: PLoS One. 2013 Aug 27;8(8):e72569. doi: 10.1371/journal.pone.0072569 (PMC3754915; doi:10.1371/journal.pone.0072569)
Supplement: Table S3 — qRT-PCR analysis of C. elegans wild-type and mutants. (DOCX) [file pone.0072569.s008.docx]

Supplemental Table S3: qRT-PCR analysis of *C. elegans* wild-type and mutants.

| ***gene of interest*** | ***wild-type*** | ***daf-11*** *–fold change in mRNA expression ± SEM* | ***pkg-1*** *–fold change in mRNA expression ± SEM* | ***pde-1,2,3,5*** *–fold change in mRNA expression* *± SEM* |
| --- | --- | --- | --- | --- |
| pkg-1 | 1 | 1.04 ± 0.097 | **0.47 ± 0.03** | 1.19 ± 0.14 |
| daf-11 | 1 | **3.49 ± 0.74^***^** | **11.55 ± 1.76^***^** | **0.21 ± 0.06^***^** |
| pde-1 | 1 | **2.38 ± 0.32^*^** | **4.93 ± 0.65^*^** | 0.61 ± 0.06 |
| pde-2 | 1 | 0.94 ± 0.06 | 0.86 ± 0.08 | 1.12 ± 0.10 |
| pde-3 | 1 | 0.55 ±0.05 | 0.58 ± 0.07 | **0.35 ± 0.45^***^** |
| pde-4 | 1 | 1.68 ± 0.19 | 2.73 ± 0.27 | 1.26 ± 0.14 |
| pde-5 | 1 | **3.57 ± 0.65^***^** | **8.52 ± 1.17^***^** | **0.15 ± 0.04^***^** |
| pde-6 | 1 | 1.07 ± 0.10 | 1.64 ± 0.16 | 0.74 ± 0.05 |

Animals were grown to adult stage (72 h) under normal conditions on NGM plates at 22°C. 500-1000 worms were harvested and processed for RNA extraction by freeze-crack and thus qRT-PCR analysis was conducted in order to determine background mutations in the cGMP-signaling pathway. Values represent means ± SEM of 3 to 8 independent RNA preparations. The fold change in mRNA expression is normalized on wild-type levels. Red: significant increase in mRNA expression compared to wild-type; green: significant decrease in mRNA expression compared to wild-type. P-values were calculated by means of ANOVA Bonferroni's multiple comparison test with ***: p-value ≤ 0.001; *: p-value ≤ 0.05.
